# Vitamin B12 Deficiency and Clinical Neuropathy with Metformin Use in Type 2 Diabetes

**DOI:** 10.3390/jox12020011

**Published:** 2022-05-31

**Authors:** Malik Dilaver Farooq, Farooq Ahmad Tak, Fauzia Ara, Samia Rashid, Irfan Ahmad Mir

**Affiliations:** 1 Department of Medicine, Government Medical College Srinagar, Srinagar 190010, India; malikdil.md@gmail.com (M.D.F.); drfarooqtak@gmail.com (F.A.T.); drirfan765742@yahoo.com (S.R.); 2 Department of Ophthalmology, Bangalore Medical College and Research Institute, Bengaluru 560002, India; fauziaara@gmail.com

**Keywords:** T2DM, metformin, vitamin B12 deficiency, clinical neuropathy

## Abstract

**Introduction**: Type 2 diabetes (T2DM), which is more prevalent (more than 90% of all diabetes cases) and the main driver of the diabetes epidemic, now affects 5.9% of the world’s adult population, with almost 80% of the total in developing countries. At present, 537 million adults (20–79 years) are living with diabetes—1 in 10. This number is predicted to rise to 643 million by 2030 and 783 million by 2045. In India, reports show that 69.2 million people are living with diabetes (8.7%) as per 2015 data. Long-term metformin treatment is a known pharmacological cause of vitamin B12 (Vit B12) deficiency, as was evident within the first 10–12 years after it started to be used. **Methods**: This was a cross-sectional study conducted in the Postgraduate Department of Medicine in one of the tertiary hospitals in Kashmir. A total of 1600 consecutive patients with T2DM were taken for the study. Out of which 700 patients met the inclusion criteria. These 700 patients were divided into two groups: those taking metformin, and those who were not on metformin. Cumulative metformin doses were recorded in patients taking metformin, using history of dose and duration of treatment. Serum Vit B12 levels were taken for all patients. Based on the results of Vit B12 levels, patients were classified into normal levels (20 pmol/L), possible B12 deficiency (150–220 pmol/l), and definite deficiency (<150 pmol/L). **Results**: Our results depicted that patients on prolonged metformin therapy showed an increase in Vit B12 deficiency by 11.16%. The prevalence of clinical neuropathy in the metformin-exposed group was 45%, whereas, a prevalence of 31.8% was found in the non-metformin group. The mean age of patients with neuropathy was higher than those without neuropathy (59.01 ± 7.14 vs. 49.95 ± 7.47) (*p*-value < 0.514, statistically insignificant). **Conclusions**: In our study, we found that metformin use is associated with Vit B12 deficiency, which is dependent upon the cumulative dose of metformin. Importantly, prolonged metformin use is also associated with an increase in the prevalence of clinical neuropathy.

## 1. Introduction

Diabetes is becoming the fastest epidemic of the 21st century. Type 2 diabetes (T2DM), which occurs in more than 90% of all diabetes cases and is the primary cause of the diabetes epidemic, now affects 5.9% of the world’s adult population, with nearly 80% of the total occurring in developing countries [1]. At present, 537 million adults (20–79 years) are living with diabetes—1 in 10. This number is predicted to rise to 643 million by 2030 and 783 million by 2045 [2]. Nowhere is the diabetes epidemic more pronounced than in India, as World Health Organization (WHO) reports show that 69.2 million people are living with diabetes (8.7%) according to data from 2015. Of these, diabetes remains undiagnosed in more than 36 million people [3]. The International Diabetes Federation (IDF) calculates the total number of diabetic subjects to be approximately 40.9 million in India; this is set to rise to 69.9 million by the year 2025 [1].

Metformin is the most commonly prescribed oral anti-diabetic drug in patients with T2DM [4]. Metformin impedes hepatic gluconeogenesis and glycogenolysis and glucose uptake from the intestines and refines peripheral insulin sensitivity. Long-term metformin treatment is a known pharmacological cause of Vit B12 deficiency, as was evident within the first 10–12 years [5,6,7,8,9] after its use [10,11]. This is clinically important because patients with diabetes often suffer from neurological symptoms, such as numbness, paraesthesia, and impaired vibration sensation and proprioception. As early as 1971, researchers began to speculate that one of the side effects of metformin use was vitamin B12 malabsorption [12]. Current research points to the effect of metformin on the calcium-dependent B12–intrinsic factor complex and absorption in the terminal ileum as the primary mechanism for Vit B12 depletion [13]. We conducted this study to determine the prevalence of vit B12 deficiency in T2DM patients, the effect of metformin therapy on vit B 12 levels, and the effect of vit B12 deficiency on neuropathy; because vit-B12-induced neuropathy is a treatable condition that may be confused with diabetic neuropathy leading to inappropriate treatment.

## 2. Methods

This was a cross-sectional study conducted in the Postgraduate Department of Medicine at Government Medical College Srinagar in North India, from September 2014 to November 2016. A total of 1600 consecutive patients of T2DM were screened for the study. Among them, 187 patients declined to participate in the study and 713 patients did not meet the inclusion criteria. A total of 700 T2DM patients participated, and were divided into two groups: those taking metformin, and those who were not on metformin. Cumulative metformin doses were recorded in patients taking metformin using their dose and duration of treatment. In all the outpatients and admitted diabetes patients, a detailed history was taken and a clinical examination was performed. Blood pressure, body mass index (BMI), HbA1c and baseline investigations were recorded in every patient. Serum Vit B12 levels were taken in all the patients. Serum samples were stored at room temperature (15–30 °C) for no longer than 7 h. All included patients were subjected to Vit B 12 assay, which was performed using the Roche E-170 Vit B12 electrochemiluminescence immunoassay (ECLIA) method [14]. Based on the results of B12 levels, patients were classified into normal levels (>220 pmol/L), possible B12 deficiency (150–220 pmol/L), and definite deficiency (<150 pmol/L) [8].

The inclusion criteria in this study were as follows: age group of 31–70 years, patients with T2DM according to American Diabetes Association, 2017 (ADA) criteria and patients withT2DM undergoing treatment for diabetes. The exclusion criteria for the study were as follows: alcoholism, ongoing pregnancy, liver disease, renal disease, thyroid disorders, history suggestive of malabsorption disorders and history of use of proton pump inhibitors & Vit B12 supplements. The baseline demographic variables, such as age, sex, dietary habits (vegetarian or on mixed diet), and HbA1c levels, were measured in both groups, as well as Vit B12 levels and severity of peripheral neuropathy (using Toronto Clinical Scoring System (TCSS) [15]. The study protocol was approved by the institutional review board and the institutional ethics committee of government medical college, Srinagar India. Verbal/written consent was obtained from each participating patients.

Data analysis was conducted using SPSS 20.0 statistical software (Statistical Package for the Social Sciences) Continuous data were summarized as mean and standard deviation. Categorical data were summarised as frequency and percentage. The difference in the prevalence of vitamin B12 deficiency and clinical neuropathy between metformin and non-metformin groups was analysed using the chi-square test. The association between cumulative metformin dose and vitamin B12 levels was analysed using Pearson’s correlation coefficient and regression analysis. Moreover, Student’s Independent *t*-test and ANOVA with post-hoc (Tukey’s Honest Significant Difference) were also employed for the analysis of data. Graphically, the data was presented using scatter diagram. A *p*-value of <0.05 was taken as statistically significant.

## 3. Results

Out of 700 T2DM patients in the age group from 30 to 70 years included in this study, 366 patients (52.29%) were males and 334 (47.71%) were females. The minimum age among patients taken in this study was 31 years and the maximum age of 70 years. The mean age of males was 50 years and that of females was 52.56 years. There were 373 (53.29%) patients in our study living in urban and 327 (46.71%) in rural areas. A total of 98% were married and only 2% were unmarried; 320 (45.71%) were literate and 380 (54.29%) were illiterate.

Out of 700 T2DM patients, 451 (64.4%) were on metformin and 249 (35.6%) patients were on insulin, sulfonylureas, and gliptins. We divided these patients into two groups: those taking metformin (metformin group) and those not taking metformin (non-metformin group). In the metformin group, 307 patients were on metformin alone, 116 patients were on metformin and sulfonylureas and 28 patients were on metformin as well as insulin. In the non-metformin group, out of 249 patients, 203 were on insulin and 46 patients were on sulfonylureas and/or voglibose. The mean BMI of the study population was 27.15 ± 2.2; for males it was 26 ± 2.31 kg/m^2^ and for females it was 28.4 ± 2.16 kg/m^2^ with a minimum value of 19.6 kg/m^2^ and maximum value of 36.8 kg/m^2^.

Based on Vit B 12 levels, the patients were divided into three groups: vit-B12-sufficient group, borderline-deficient group and B12-deficient group.

The patients were divided into two groups, metformin and non-metformin, with a mean treatment duration as given in Table 1.

**Figure 1:**
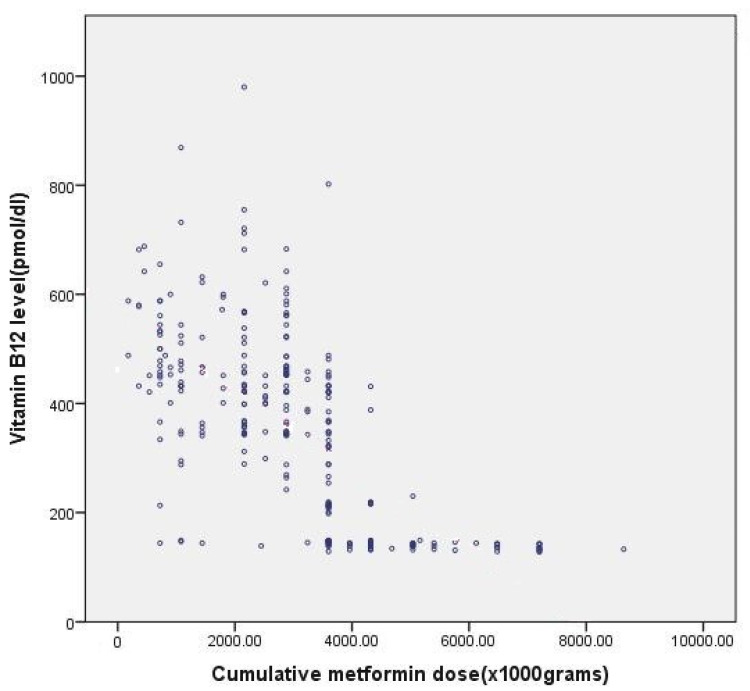
(Scatter diagram): Relationship between cumulative dose of metformin and B12 deficiency. (Pearson Correlation coefficient is −0.6 and *p* valve < 0.001).

The relationship between cumulative dose of metformin and vit B12 deficiency is shown in Table 2 and Figure 1.

Table 2 suggests that the cumulative dose of metformin, rather than duration of metformin therapy, is more strongly associated with vitamin B12 deficiency. In Figure 1, Pearson’s correlation coefficient for cumulative dose of metformin and observed vitamin B12 levels was −0.66, which is statistically significant (*p* value < 0.001). This clearly shows that, with an increase in cumulative dose of metformin, vitamin B12 levels decrease.

As we can see from Table 3, all these intervals contain 0.00; therefore, we conclude that all three groups are no different from each other.

The relationship between neuropathy score (Toronto clinical scoring system (TCSS)) and metformin use is shown in Table 4.

## 4. Discussion

We defined definite and possible (borderline) deficiency as serum vit B12 levels of <150 and <220 pmol/L, respectively [8,16,17,18,19,20]. In adults, a vit B12 level of 150 pmol/L is considered the lowest level for an adequate state. In a developing deficiency, serum concentrations are maintained by depleting body storage. Therefore, a concentration of 150 pmol/L might not reflect a sufficient vit B12 status, [21] and a cut-off value of <220 pmol/L is proposed by some [20]. Anaemia tends to occur only when metabolic deficiency is moderately severe [22] or the deficiency is severe enough to affect the haematological indices. In addition, macrocytosis can be masked by coexisting microcytic processes, including thalassaemia and iron deficiency [23]. Estimation of vit B12 deficiency in T2DM patients taking metformin will help in the formulation of guidelines regarding vit B12 monitoring and supplementation in such patients. The aim of our study was to identify the prevalence of vit B12 deficiency in T2DM patients, to observe the effects of metformin on vit B 12 levels and the effects of vit B12 deficiency on neuropathy, as vit-B12-induced neuropathy is a treatable disease that may be confused with diabetic neuropathy, hence leading to inappropriate management. Seven hundred patients who fulfilled the criteria for T2DM as framed by ADA were enrolled in this study.

Out of 700 T2DM patients, 451 (64.4%) and 249 (35.6%) patients belonged to the metformin and non-metformin group, respectively (Table 1). The mean duration of diabetes in the study population from the time of diagnosis was 63.21 ± 29.23 months, with 62.5 ± 28.6 months for males and 64.01 ± 27.9 months for females. These parameters matched a study conducted by Singh AK et al. [24].

The prevalence of vit B12 deficiency in the metformin and non-metformin observed groups was 33.26% and 22.1%, respectively. This shows that patients on prolonged metformin therapy showed an 11.16% increase in vit B12 deficiency. A study conducted in Indian population by Singh A K et al. [24] found that mean serum B12 levels were significantly lower in the metformin-exposed group (*n* = 84) compared with the non-metformin-exposed group (*n* = 52) (410 ± 230.7 versus 549.2 ± 244.7, *p* = 0.0011), but another study conducted by Gupta et al. [25], found a negative correlation between the duration of metformin use and vitamin B12 levels (r = −0.40). Raheel Iftikhar et al. [26] found that serum B12 levels were found to be low in 35 patients (31%) on metformin, as compared to only nine patients (8.6%) among controls, (*p* value 0.002). Mean B12 levels were significantly lower in the metformin group: 311 pg/mL (±194.4), *p* value 0.03. A study by Omar Marar et al. [27] showed that 71 {33%} patients on metformin had vitamin B12 deficiency, compared to only 5 {7.5%} in the control group {*p* < 0.00001}, and Joline WJ Beulens et al. [28] also showed that the prevalence of cobalamin deficiency was 28.1%, while a holotranscobalamin deficiency occurred in 3.9% of patients’ taking metformin.

In our study, the mean cumulative dose of patients on metformin was 2999.8 ± 1606.3 g, with a maximum dose of 8640 g and a minimum dose of 180 g. We found that, with an increase in the cumulative dose of metformin, vitamin B12 levels decreased. The cumulative dose of metformin in vitamin-B12-deficient patients was 4663.2 ± 1506.8, whereas in the borderline-vitamin-B12-deficient group, it was 3637.89 ± 776.71, and in patients with normal vitamin B12 levels, the cumulative dose was 2230.32 ± 1051.79 (*p*-value < 0.001). Pearson’s correlation coefficient for a cumulative dose of metformin and the observed vitamin B12 levels was −0.662, which is statistically significant (*p* value < 0.001). This clearly shows that, with an increase in the cumulative dose of metformin, the vitamin B12 levels decrease (Table 2 and Figure 1). These observations are supported by previous studies conducted by Joline W J Beulens et al. [28], Singh AK et al. [24], Shihong Chen, et al. [29].

The prevalence of neuropathy in patients in the present study is 40.43% according to the Toronto Clinical Scoring System. The prevalence of clinical neuropathy in the metformin-exposed group was 45%, whereas a prevalence of 31.8% was found in the non-metformin group. The mean age of patients with neuropathy was higher than those without neuropathy (59.01 ± 7.14 vs. 49.95 ± 7.47) (*p*-value < 0.514, statistically insignificant) (Table 5 and Table 6). These results are consistent with studies conducted by Shihong Chen et al. [29], Muhammad Umer Nisar et al. [30] and Yacoub G. Bahou et al. [31].

The data from our study showed that, with an increase in the dose of metformin, the neuropathy scores performed by TCSS increased, which suggests that neuropathy worsens as the cumulative dose of metformin increases. The mean TCSS scores of the whole study population, for metformin-exposed and non-metformin groups, were 5.91 ± 2.997, 6.36 ± 3.43 and 5.1 ± 3.88, respectively (Table 4). According to linear regression analysis, metformin use was shown to worsen the clinical score of neuropathy (coeff. = −2.947, *p*-value < 0.001) (Table 7). However, HbA1c levels in patients with and without clinical neuropathy were 8.7 ± 1.27 and 7.92 ± 0.88, respectively (*p*-value < 0.001), showing that HbA1c levels in the clinical neuropathy group were higher, by a mean of 0.78%, than those in patients without neuropathy (Table 8). This suggests that poor glycemic control is also associated with worsed diabetic neuropathy status. This finding needs to be thoroughly studied; whether metformin use further adds to clinical neuropathy in diabetes by causing vitamin B 12 deficiency. Similar results were obtained in a study conducted by Yacoub G. Bahou et al. [31], Diabetes Control and Complication Trial (DCCT) [32], UK Prospective Diabetes Study Group [33], Kumamoto trial [34] and Huang et al. [35].

## 5. Limitations

The major limitation of our study is that it was a cross-sectional study, and we could not find any evidence of a temporal relationship between exposure and outcome. We did not include patients with vitamin B12 supplements, which may have helped us more with treatment suggestions.

## 6. Conclusions

In our study, we found that metformin use is associated with vitamin B12 deficiency, depending on the cumulative metformin dose. Prolonged metformin use is associated with an increase in the prevalence of clinical neuropathy, possibly due to vitamin B12 deficiency caused by metformin use. Poor glycemic control is also associated with an increased prevalence of clinical neuropathy. Therefore, the rationale is to screen patients who are to undergo metformin therapy for vitamin B12 deficiency and monitor for B12 deficiency once the patient has been started on metformin.

## Figures and Tables

**Table 1:** Two groups with mean treatment duration.

	Number	Duration of Treatment (Months)
Metformin Group	451	56 ± 5.9
Non-metformin group	249	76 ± 6.5

**Table 2:** Relationship between cumulative dose of metformin (grams {g}) and Vit B12 (pmol/l) deficiency.

B12 Deficiency (pmol/L)	No. of	Mean (g)	SD	Min. (g)	Max. (g)	*p* Value
	Patients					
Vitamin BI2	205	4663	1506.8	720	8640	
Deficient (<150)						
Borderline	42	3637.9	776.7	720	4320	<0.001
Vitamin BI2						
Deficient (150–220)						
Vitamin BI2	204	2230.3	1051.8	180	5040	
Sufficient (>220)						
**Total**	**451**	**2999.8**	**1606.3**	**180**	**8640**	

**Table 3:** Post-hoc (Tukey’s Honest Significant Difference) for intergroup comparison of patients on metformin.

B12 Deficiency		Sig.
Yes	Borderline	0.002
	No	0.000
Borderline	Yes	0.002
	No	0.000
No	Yes	0.000
	Borderline	0.000

**Table 4:** Relationship between neuropathy score (Toronto clinical scoring system (TCSS)) and metformin use.

Group	TCSS Score	*p*-Value
All patients	5.9 ± 2.9	
Metformin-exposed	6.3 ± 3.4	<0.001
Non-Metformin group	5.1 ± 3.9	

**Table 5:** Prevalence of clinical neuropathy in different age groups.

			Neuropathy		*p*-Value
			Yes	No	
Age	31–40	No. of Patients	7	46	
		Percentage	2.5%	11%	
	41–50	No. of Patients	36	220	0.5 (NS)
		Percentage	12.7%	52.9%	
	51–60	No. of Patients	121	114	
		Percentage	42.6%	27.4%	
	61–70	No. of Patients	120	36	
		Percentage	42.2%	8.7%	
Total		No. of Patients	284	416	
		Percentage	100.0%	100.0%	

**Table 6:** Prevalence of clinical neuropathy in different groups.

	No. of Patients	Percentage	*p*-Value
Neuropathy in the study population	283/700	40.4	0.001
Neuropathy in the metformin group	203/451	45.0	
			(Sig.)
Neuropathy in Non-metformin group	80/249	31.8	

**Table 7:** Linear regression analysis, using TCSS score as a dependent factor and metformin use (yes/no) and duration of t2dm as independent factors.

Coefficients							
	Unstandardized		Standardized			95.0% Confidence	
	Coefficients		Coefficients	t	*p*-Value	Interval for B	
	B	SE	Beta			Lower	Upper
						Bound	Bound
(Constant)	3.7	0.2		15.0	<0.001	3.2	4.2
Metformin	−2.9	0.1	−0.5	−15.4	<0.001	−3.3	−2.6
Use							
Duration of	0.09	0.003	0.9	31.1	<0.001	0.091	0.1
Diabetes							
(months)							
		Dependent Variable: TCSS Score Using linear regression analysis, it was observed that metformin use worsened the clinical neuropathy score (coeff. = −2.947, *p*-value < 0.001)					

**Table 8:** Relationship between HbA1c level and clinical neuropathy.

Neuropathy	No. of Patients	Mean HbA1c	Std.	*p*-Value
		(%)	Deviation	
Present	284	8.7	1.2	<0.001
Absent	416	7.9	0.9	

## Data Availability

Not applicable.
